# Mutation of the Thyroid Hormone Receptor Beta Gene (*THRB*) Causes Vitelliform Macular Dystrophy with High Intrafamilial Variability

**DOI:** 10.3390/genes16101240

**Published:** 2025-10-20

**Authors:** Elisa A. Mahler, Lars C. Moeller, Katharina Wall, Marlene Saßmannshausen, Bettina Kron, Hanno J. Bolz, Frank G. Holz, Philipp Herrmann

**Affiliations:** 1 Department of Ophthalmology, University Hospital Bonn, 53127 Bonn, Germany; elisa.mahler@ukbonn.de (E.A.M.); katharina.wall@ukbonn.de (K.W.); marlene.sassmannshausen@ukbonn.de (M.S.); frank.holz@ukbonn.de (F.G.H.); 2 Ophthalmology Department, Necker Enfants Malades University Hospital, Assistance Publique-Hôpitaux de Paris (AP-HP), Paris Cité University, 75015 Paris, France; 3 Department of Endocrinology, Diabetes and Metabolism, Division of Laboratory Research, University Hospital Essen, 45147 Essen, Germany; lars.moeller@uk-essen.de; 4 Bioscientia Human Genetics, Institute for Medical Diagnostics GmbH, 55218 Ingelheim, Germany; bettina.kron@bioscientia.de (B.K.); hanno.bolz@bioscientia.de (H.J.B.)

**Keywords:** inherited retinal dystrophies (IRD), thyroid hormone receptor beta gene (*THRB*), macular dystrophy, interfamily variability, phenotype–genotype correlation, phenotype expansion

## Abstract

Background/Objectives: Herein, we report the clinical cases of two affected first-degree relatives from a family with highly variable macular dystrophy, expanding the known phenotype spectrum with mutations in the thyroid hormone receptor beta gene (*THRB*). Methods: Multimodal retinal imaging included wide-field fundus photography, fundus autofluorescence (FAF), spectral domain optical coherence tomography (SD-OCT) imaging, performed alongside functional testing (visual fields, electroretinogram (ERG)), metabolic blood analyses, and genetic testing of both cases. Results: A 67-year-old female patient presenting with reading difficulties and visual impairment since childhood was referred for evaluation and counseling for potential treatment options. Extensive ophthalmologic examination, including multimodal retinal imaging and functional testing, revealed an occult macular dystrophy. Her 39-year-old son reported similar visual symptoms in combination with mild photophobia. In multimodal retinal imaging, he also showed a macular dystrophy but with a vitelliform phenotype. Genetic testing identified the heterozygous pathogenic variant c.283+1G>A in the thyroid hormone receptor beta gene (*THRB*) in both patients. Conclusions: This report shows a high intrafamilial variability of macular dystrophy caused by a heterozygous *THRB* mutation, which has only recently been recognized as a cause of macular dystrophy. Here, we describe a novel clinical presentation characterized by a vitelliform lesion, expanding the phenotypic spectrum of *THRB*-associated macular dystrophy.

## 1. Introduction

Inherited retinal diseases (IRDs) are a phenotypically and genetically heterogeneous group of monogenic diseases leading to visual impairment with currently more than 300 identified causative genes (RetNet database: https://retnet.org/summaries#a-genes, accessed on 3 July 2025). A subgroup of IRDs involves primary degeneration of the cone photoreceptors, which are densely concentrated in the macula, causing various types of macular dystrophies, e.g., Stargardt disease or cone- and rod-dystrophy. About 50 genes are associated with cone-dominated diseases [1]. IRDs are further categorized into non-syndromic forms, affecting only the retina, and syndromic forms, which involve additional systemic manifestations [2,3,4].

One form of cone-dominated IRD is associated with variants in the thyroid hormone receptor beta gene (*THRB*). *THRB* encodes the thyroid hormone receptor beta (TRβ), which is expressed in two isoforms that differ in their N-termini, TRβ1 and TRβ2. TRβ1 is expressed in the retina and anterior eye tissues, such as the iris and ciliary body, but also in several other tissues [5], whereas TRβ2 is restricted to the hypothalamus, pituitary, cochlea, and retina [6]. TRβ2 regulates the hypothalamus–pituitary–thyroid axis and, consequently, the concentrations of TSH and the thyroid hormones (triiodothyronine, T3, and thyroxine, T4), which also regulate the specification of the three different cone subtypes [7].

*THRB* mutations are usually associated with autosomal dominant resistance to thyroid hormone beta (RTHβ) [8]. Such mutations typically affect the ligand-binding domain and adjacent hinge region of TRβ1 and 2. Interestingly, two recently reported variants in the *THRB* gene are associated with autosomal dominant macular dystrophy. The *THRB* variant c.283+1G>A is located in the donor splice site at the end of the N-terminal TRβ1 exclusive exon 4 [9] and should therefore not affect the TRβ2 isoform. In 2023, Fernández-Suárez et al. described the association with a macular dystrophy as the major clinical feature in three Spanish families [10]. Recently, in 2025, Fernández-Caballero et al. described two more Spanish families with the same c.283+1G>A variant and reported a second pathogenic splicing variant c.283G >A, affecting the same splice donor site, in another Spanish family [11]. The reported patients with *THRB* variants showed great variability in the age of onset and severity of symptoms, but exhibited similar macular changes on retinal imaging, including foveal cavitation and ellipsoid zone disruption on OCT scans.

Here, we describe a novel ophthalmic phenotype with a vitelliform pattern and a high intrafamilial variability in patients carrying the *THRB* c.283+1G>A mutation.

## 2. Materials and Methods

Subjects were identified at a tertiary referral center for IRDs at the Department of Ophthalmology, University Hospital Bonn, in Germany. Written informed consent was obtained from the participants in accordance with institutional and ethical guidelines. After obtaining a general medical and ophthalmological history, each subject underwent a standardized and detailed clinical examination, including best-corrected visual acuity testing (BCVA), visual field testing (Octopus 900 and mesopic microperimetry, S-MAIA, Icare, Padua, Italy), multimodal retinal imaging, full-field mesopic and scotopic electroretinography (ERG), anterior segment, and fundus examination in dilated pupils. The standardized multimodal retinal imaging protocol included spectral domain optical coherence tomography (SD-OCT with a horizontal and vertical line scan through fovea, a 61 b-scans volume 30° × 25°, automated real-time (ART) minimum 10, High-speed mode, Spectralis, Heidelberg Engineering, Heidelberg, Germany), 55° fundus autofluorescence (FAF) with blue excitation light (Spectralis, HRT+OCT, ART minimum 80, Heidelberg Engineering, Heidelberg, Germany) and wide-field fundus photography (Clarus 500, ZeissMeditec, Oberkochen, Germany). Genetic testing of the index patient was performed using PacBio SMRTbell prep kit 3.0 (Pacific Biosciences of California, Inc. (“PacBio”), Menlo Park, CA, USA) for long-read whole-genome sequencing (LR-WGS) on a PacBio Revio^®^ system (Pacific Biosciences of California, Inc. (“PacBio”), Menlo Park, CA, USA), as previously reported (PMID: 40170356), at an average coverage of 26,4-fold. In brief, LR-WGS data were processed with an in-house bioinformatic pipeline (implicating tools to assess functional relevance, conservation, and splice effects). Identified variants were compared to the literature, external and internal allele frequency databases, and mutation databases such as the Human Gene Mutation Database (HGMD) and ClinVar. Segregation analysis in the affected son was carried out by PCR amplification and Sanger sequencing of the respective *THRB* exon with its adjacent splice sites.

Clinical data relevant to the disease of each patient were obtained, including metabolic blood testing with serum thyroid hormone profiling (triiodothyronine, [T3], thyroxine, [T4], and thyroid-stimulating hormone, [TSH]).

## 3. Results

A 67-year-old female patient (III:5) was referred for evaluation and counseling for potential treatment options. She complained of reading difficulties and visual impairment despite wearing glasses since the age of 53 and recent bilateral cataract surgery. No syndromic findings and, in particular, no endocrine disorders were reported. She was euthyroid with TSH and FT4 within the reference range (TSH 2.15 mU/L [0.55–4.78], FT4 17.0 pmol/L [11.5–22.7]). Detailed patient characteristics are given in Table 1.

**Figure 1:**
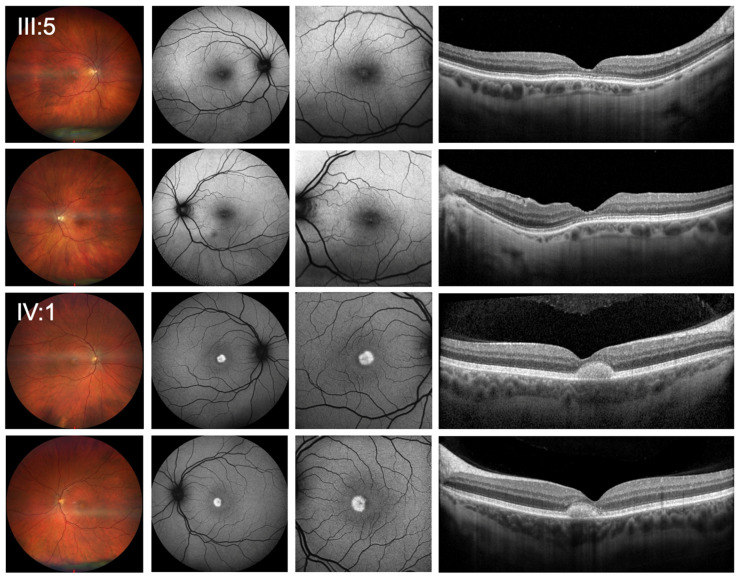
Clinical presentation of patients. Wide-field color fundus photography (first column), 55 degrees blue-light fundus autofluorescence (second column), 30 degrees blue-light fundus autofluorescence imaging (FAF) (third column) as well as the horizontal spectral-domain optical coherence tomography line scan (SD-OCT) (last column) showed mild bilateral central changes with disruptions of the foveal ellipsoid zone for the index patient (III:5) and bilateral vitelliform lesions in the central macula, which were hyperautofluorescent in FAF and correlate with subretinal deposits without signs of fluid accumulation or hypertransmission in SD-OCT for her son (IV:1).

Best-corrected visual acuity (BCVA) was 0.5 logMAR (0.32 Snellen decimal) in the right eye (OD) and 0.4 logMar (0.4 Snellen decimal) in the left eye (OS). Objective refraction showed −2.75/−1.25 × 166° for OD and −2.75/−0.75 × 43° for OS. Ophthalmologic examination at initial presentation revealed normal pseudophakic anterior segments in both eyes and mild bilateral central changes with disruptions of the foveal ellipsoid zone on SD-OCT imaging (Figure 1).

**Figure 2:**
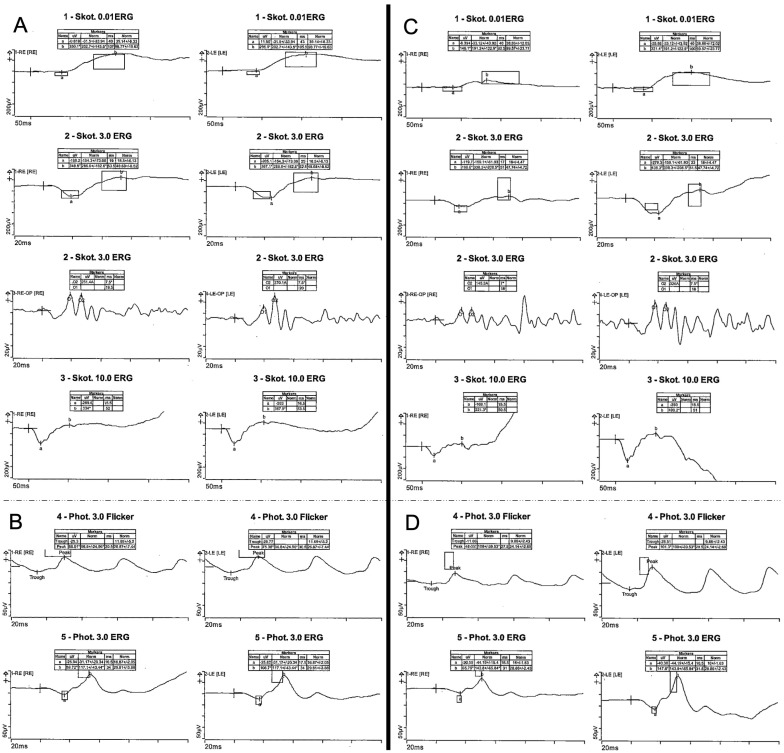
Electrophysiology testing. Full-field electrophysiology testing (ERG) of a 67-year-old index patient (III:5) with normal scotopic (**A**) and photopic (**B**) responses and ERG of her 39-year-old son (IV:1) with normal scotopic (**C**) and pathologic photopic (**D**) responses with reduced amplitudes.

Detailed functional testing showed normal photopic and scotopic responses on full-field ERG (Figure 2).

**Figure 3:**
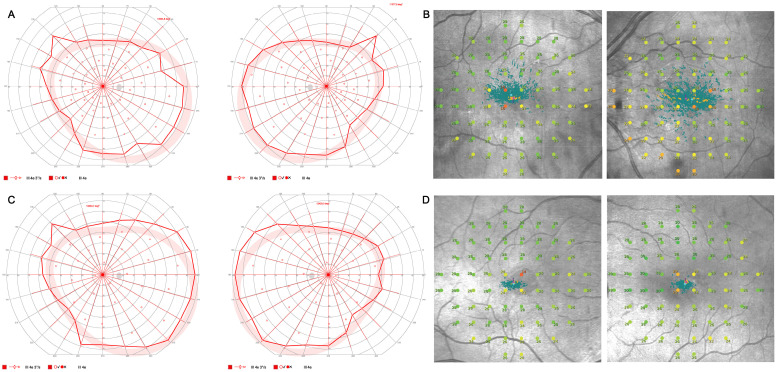
Visual field testing. Visual field testing with normal outer visual field borders (bilateral 80° temporal, 60° nasal, 45° superior, 65° inferior) in Octopus 900 (III 4e) (**A**) and central restriction with decreased fixation stability (Fixation loss right eye (OD) 14%, left eye (OS) 25%) on mesopic microperimetry (**B**) of a 67-year-old index patient (III:5) and visual field testing of her son (IV:1) with normal outer visual field borders (bilateral 90° temporal, 55° nasal, 45° superior, and 65° inferior) in Octopus 900 (III 4e) (**C**) and mild central restriction with preserved fixation stability (Fixation loss bilateral 0%) on mesopic microperimetry (**D**).

Visual field testing revealed a pathological central visual field in both eyes with normal outer visual field borders (bilateral 80° temporal, 60° nasal, 45° superior, and 65° inferior) in Octopus 900 (III 4e) but central restriction with decreased fixation stability (Fixation loss OD 14%, OS 25%) on mesopic microperimetry (Figure 3).

The 39-year-old son (IV:1) reported similar visual symptoms in combination with mild photophobia, particularly during summer. He noted long-standing poor visual acuity, although he had successfully passed the visual requirements for a driver’s license. No syndromic findings and, in particular, no endocrine disorders were reported. Specifically, he was euthyroid with TSH and FT4 within the reference range (TSH 0.86 mU/L [0.55–4.78]), FT4 15.2 pmol/L [11.5–22.7] (Table 1).

At his initial presentation, BCVA was 0.3 logMAR (0.5 decimal Snellen) for OD and 0.2 logMAR (0.63 decimal Snellen) for OS. Objective refraction showed −1.50/−1.25 × 7° for OD and −1.25/−0.75 × 177° for OS. Slit lamp examination revealed no anterior segment abnormalities. Funduscopic examination showed bilateral vitelliform lesions in the central macula, which were hyperautofluorescent in FAF. In SD-OCT, the lesions correlated with subretinal deposits without signs of fluid accumulation or hypertransmission (Figure 1). Functional testing showed pathologic photopic and normal scotopic responses on full-field ERG with reduced amplitudes (Figure 2) and pathological central visual field in both eyes with normal outer visual field borders (bilateral 90° temporal, 55° nasal, 45° superior, 65° inferior) in Octopus 900 (III 4e) but mild central restriction with preserved fixation stability (Fixation loss bilateral 0%) on mesopic microperimetry (Figure 3).

**Figure 4:**
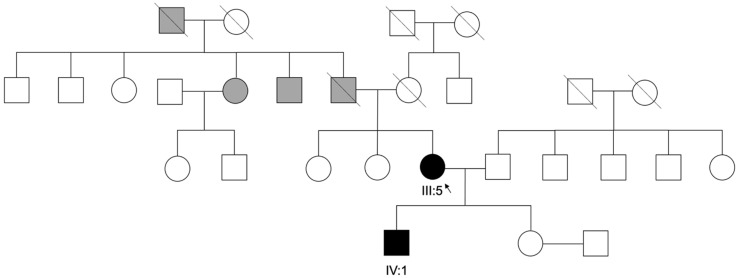
Family Pedigree. Pedigree and segregation of a pathogenic *THRB* variant c.283+1G>A, in the index patient and her son (III:5, IV:1). Gray symbols represent individuals with uncertain clinical diagnosis and white symbols represent unaffected individuals.

Regarding family history, no consanguinity was reported. The family pedigree is provided in Figure 4.

Molecular genetic testing identified a heterozygous variant c.283+1G>A in the *THRB* gene, which was recently described and classified as pathogenic. At the time of analysis, the retinal dystrophy panel used comprised 231 genes. In addition, genes listed under the following HPO terms were evaluated: HP:0000608: Macular degeneration, HP:0007754: Macular dystrophy, HP:0000548: Cone/cone-rod dystrophy, HP:0000556: Retinal dystrophy, HP:0001103: Abnormal macular morphology, HP:0000479: Abnormal retinal morphology, HP:0000546: Retinal degeneration. A search based on specific keywords was conducted on a scientific basis. It was only during this search that the variant in the *THRB* gene was identified. Apart from carrier status, no other pathogenic variants or variants of uncertain significance for the specified indication could be identified.

After detecting the *THRB* genotype in the female patient, we offered the patient’s son to present to our clinic for further workup. Molecular testing revealed the familial pathogenic *THRB* variant c.283+1G>A in the *THRB* gene in a heterozygous state (Table 2).

## 4. Discussion

We report a novel ophthalmic phenotype associated with the variant c.283+1G>A in the *THRB* gene, characterized by a vitelliform pattern and a high inter- and intrafamilial variability. This expands the known clinical spectrum of *THRB*-associated macular dystrophies.

The first IRD cases associated with the novel pathogenic variant c.283+1G>A in *THRB* have been reported by Fernández-Suárez et al. in 2023 [10] and subsequently by Fernández-Caballero et al. in 2025 [11]. Both groups mentioned extensive variability of age of onset, from early childhood to 65 years. Nevertheless, most patients showed similar findings on fundus imaging, OCT, and FAF, which appeared to be independent of age and severity of symptoms [11]. They described macular atrophy, orange/yellow lesions in the fovea, bright yellow/orange distributed spots consistent with lipofuscin deposits, mild subfoveal retinal detachment in one patient, and chorioretinal atrophy with hypertrophy of the RPE in another patient [10] and disruption of the subfoveal ellipsoid layers [11]. In our study, the female patient (III:5) presented with clinical features consistent with previous reports. However, her son exhibited a new specific phenotype with a vitelliform pattern with hyperreflective subfoveal deposits in SD-OCT and a central hyperautofluorescence in FAF. The genes known to cause vitelliform lesions are limited and include *BEST1* (OMIM#153700), *PRPH2* (OMIM#608161), *IMPG1* (OMIM#616151), and *IMPG2* (OMIM#616152). Although the index patient’s son is the only reported individual with *THRB*-related macular dystrophy and vitelliform lesions, and as a two-case report, broader generalizations are limited, we suggest that *THRB* should be considered as another gene whose mutations may elicit a vitelliform phenotype.

*THRB* mutations typically lead to RTHβ, a rare disease characterized by increased concentrations of fT4 together with normal or increased concentrations of TSH. Most patients are considered clinically euthyroid and present a wide phenotypic variability [12]. Visual impairment and cone dysfunction have been described in rare cases of RTHβ [13]. However, none of those patients met the clinical criteria for macular dystrophy, Stargardt disease, or cone dystrophy [13].

The c.283+1G>A mutation of a splice site may lead to skipping of TRβ1 exon 4, resulting in a deletion of 87 amino acids of the N-terminus (p.Glu8_Lys94del) or the deletion of 2 amino acids and a substitution (p.Cys93_Gly95delinsTrp) [11]. This N-terminal mutation and the predicted changes in the TRβ1 protein differ from the mutations found in RTHβ, which typically affect the ligand-binding domain or the hinge region of TRβ. As TRβ2 remains unaffected, it is reasonable that Fernández-Suárez et al. reported that none of the affected individuals received a clinical diagnosis of RTHβ. Most patients were euthyroid, and none showed the typical combination of elevated T4 with normal or elevated TSH. However, potentially related features such as goiter, hyperlipidemia, or hearing impairment were observed, and two patients showed elevated TSH levels [10]. Two years later, Fernández-Caballero et al. reported no observation of endocrine disorders in their patients, and the hormone tests (including TSH, T3, and T4 levels) showed normal results [11]. In agreement with these reports, we observed normal results of the hormone testing in our female patient (III:5). The mechanism of N-terminal TRβ1 mutations leading to autosomal dominant macular dystrophy is probably different from that of ligand-binding domain mutations and has yet to be determined.

## 5. Conclusions

In conclusion, we described a novel clinical presentation with a vitelliform pattern, expanding the phenotype and underlining the high intra- and interfamilial variability of *THRB*-associated macular dystrophy. *THRB* should therefore be included in the targeted panel sequencing of IRD patients, especially those with autosomal dominant inheritance.

## Figures and Tables

**Table 1:** Patient characteristics. Ophthalmologic data of patients. Abbreviations: OD, right eye; OS, left eye; BCVA, best-corrected visual acuity; FAF, fundus autofluorescence; SD-OCT, spectral domain optical coherence tomography; ERG, electroretinography.

Patient ID	Gender	Age at Onset (Years)	Age at Time of Evaluation (Years)	First Symptoms	Other Visual Symptoms	Refraction [dpt] (sph/cyl) OD/OS	BCVA OD/OS logMAR/Snellen Decimal	Ophthalmologic Examination				Clinical Diagnosis	Extraocular Manifestations
								Fundus Images and FAF	SD-OCT	Electrophysiology	Perimetry		
III:5	Female	53	67	Visual impairment	Reading difficulties	−2.75/−1.25 −2.75/−0.75	0.5/0.32 0.4/0.4	Mild macular alterations with a granular appearance	Disruption of subfoveal ellipsoid layers	Full-field ERG: Normal limits	Normal outer visual field borders, central restriction	Occult macular dystrophy	None
IV:1	Male	Child-hood	39	Visual impairment	Photophobia in summer	−1.50/−1.25 −1.25/−0.75	0.3/0.5 0.2/0.63	Bilateral orange round lesions in the macula, central hyperautofluorescence	Vitelliform pattern with hyperreflective subfoveal deposition	Full-field ERG: pathologic photopic responses with reduced amplitudes, normal scotopic response	Normal outer visual field borders, central restriction	Vitelliform macular dystrophy	None

**Table 2:** Genetic characteristics. Genetic data of patients. Abbreviations: *THRB*, thyroid hormone receptor beta gene; TRβ1, thyroid hormone receptor beta 1.

Patient ID	Gene	Zygosity	Nucleotide	Class	Protein	Reference
III:5	*THRB* (*NM_000461.5*)	heterozygous	c.283+1G>A	pathogen	Disruption of a splice site affecting the TRβ1 isoform	Fernández-Suárez et al., 2023 [10] Fernández-Caballero et al., 2025 [11]
IV:1	*THRB* (*NM_000461.5*)	heterozygous	c.283+1G>A	pathogen	Disruption of a splice site affecting the TRβ1 isoform	Fernández-Suárez et al., 2023 [10] Fernández-Caballero et al., 2025 [11]

## Data Availability

The raw data supporting the conclusions of this article will be made available by the authors on request.
